# Modularized bioceramic scaffold/hydrogel membrane hierarchical architecture beneficial for periodontal tissue regeneration in dogs

**DOI:** 10.1186/s40824-022-00315-0

**Published:** 2022-12-02

**Authors:** Yingming Wei, Zhongxiu Wang, Jiayin Han, Xiaojian Jiang, Lihong Lei, Xianyan Yang, Weilian Sun, Zhongru Gou, Lili Chen

**Affiliations:** 1 Department of Oral Medicine, the Second Affiliated Hospital, School of Medicine, Zhejiang University, Jiefang Road 88#, Hangzhou, 310009 People’s Republic of China; 2 Bio-Nanomaterials and Regenerative Medicine Research Division, Zhejiang-California International Nanosystems Institute, Zhejiang University, Hangzhou, 310058 People’s Republic of China

**Keywords:** Biphasic hierarchical architecture, Modularized functions and structures, Bioactive scaffolds, Hydrogel membrane, Guided tissue regeneration

## Abstract

**Background:**

Destruction of alveolar bone and periodontal ligament due to periodontal disease often requires surgical treatment to reconstruct the biological construction and functions of periodontium. Despite significant advances in dental implants in the past two decades, it remains a major challenge to adapt bone grafts and barrier membrane in surgery due to the complicated anatomy of tooth and defect contours. Herein, we developed a novel biphasic hierarchical architecture with modularized functions and shape based on alveolar bone anatomy to achieve the ideal outcomes.

**Methods:**

The integrated hierarchical architecture comprising of nonstoichiometric wollastonite (nCSi) scaffolds and gelatin methacrylate/silanized hydroxypropyl methylcellulose (GelMA/Si-HPMC) hydrogel membrane was fabricated by digital light processing (DLP) and photo-crosslinked hydrogel injection technique respectively. The rheological parameters, mechanical properties and degradation rates of composite hydrogels were investigated. L-929 cells were cultured on the hydrogel samples to evaluate biocompatibility and cell barrier effect. Cell scratch assay, alkaline phosphatase (ALP) staining, and alizarin red (AR) staining were used to reveal the migration and osteogenic ability of hydrogel membrane based on mouse mandible-derived osteoblasts (MOBs). Subsequently, a critical-size one-wall periodontal defect model in dogs was prepared to evaluate the periodontal tissue reconstruction potential of the biphasic hierarchical architecture.

**Results:**

The personalized hydrogel membrane integrating tightly with the nCSi scaffolds exhibited favorable cell viability and osteogenic ability in vitro, while the scratch assay showed that osteoblast migration was drastically correlated with Si-HPMC content in the composite hydrogel. The equivalent composite hydrogel has proven good physiochemical properties, and its membrane exhibited potent occlusive effect in vivo; meanwhile, the hierarchical architectures exerted a strong periodontal regeneration capability in the periodontal intrabony defect models of dogs. Histological examination showed effective bone and periodontal ligament regeneration in the biomimetic architecture system; however, soft tissue invasion was observed in the control group.

**Conclusions:**

Our results suggested that such modularized hierarchical architectures have excellent potential as a next-generation oral implants, and this precisely tuned guided tissue regeneration route offer an opportunity for improving periodontal damage reconstruction and reducing operation sensitivity.

**Graphical Abstract:**

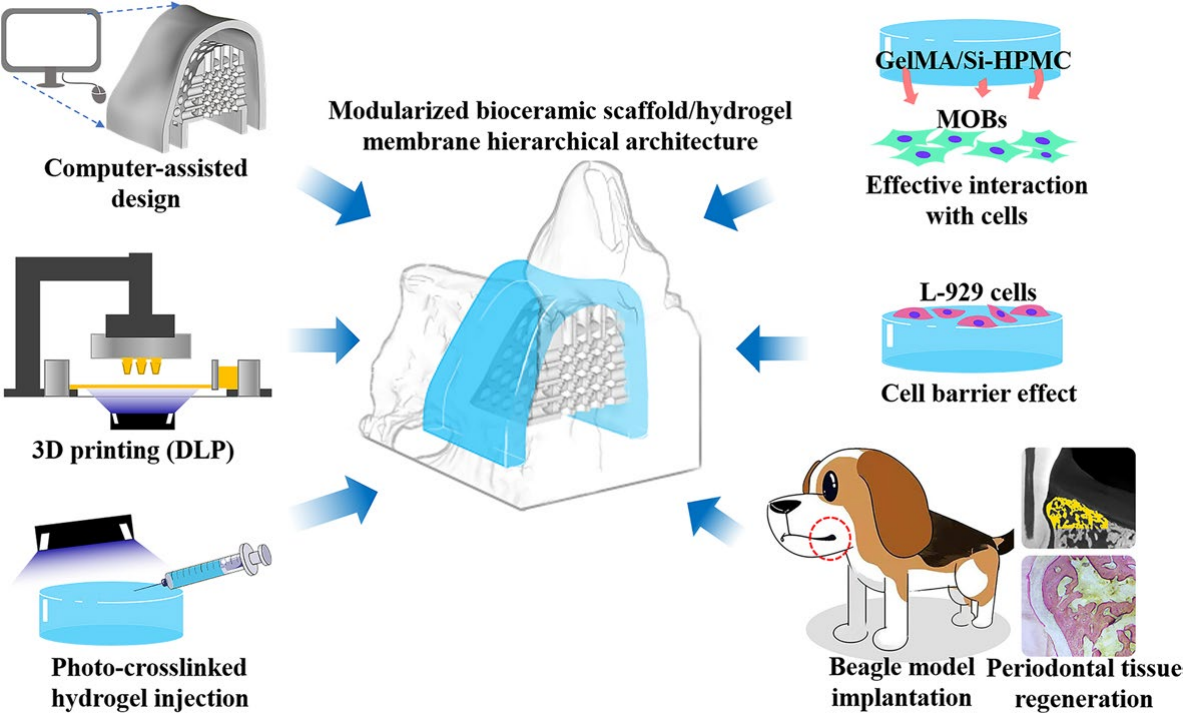

## Introduction

Periodontal disease (PD) is a typical chronic inflammatory response which is characterized by destruction of periodontium, affecting 20–50% of the population worldwide [1]. PD is usually caused by a combination of micro-organisms and a predisposing host response which leads to the destruction of gingiva, alveolar bone, periodontal ligament and cementum [2]. Initial therapy for PD consists of scaling and root planning to remove the causes of the disease (e. g. supragingival and subgingival dental plaque and calculus) [3, 4]. Local delivery of drugs, systemic antibiotics and systemic host modulation agents are also proposed as adjunctive therapy to enhance treatment outcomes [3]. Such non-surgical periodontal therapies are effective in eliminating inflammation and resulting in the formation of some new attachment [5]. However, surgical access is still required to restitution and integrum the reduced periodontium and residual deep intrabony defects [6, 7].

Nowadays, guided tissue regeneration (GTR) is known as a common method to reconstruct the lost function and construction of periodontal tissue [8]. After surgical exposure and debridement of a periodontal defect, different types of cells can repopulate the wound, including gingival epithelial cells, gingival connective tissue cells, bone cells, and periodontal ligament (PDL) cells [6]. When the root surface was recolonized by epithelial cells, the attachment of long junctional epithelium is formed. Long junctional epithelium is attached to root surfaces by hemidesmosomes which are vulnerable to dental plaque and inflammatory response [4]. Previous studies also revealed that root resorption might occur when cells derived from the gingival connective tissue were proliferating to contact with the root surface [9]. In contrast, cells from the periodontal ligament and alveolar bone can migrate into the wound site and support the formation of alveolar bone and new periodontal attachment [10]. And as such, a physical barrier membrane is used in GTR surgery to prevent the migration of oral epithelium and gingival connective tissue to root surface; meanwhile a sheltered space may maintain in the defect area for reconstruction of hierarchical structures including bone tissue, cementum, and periodontal ligament [11, 12]. Accordingly, the barrier membrane should exhibit soft tissue cell occlusion and alveolar defect space maintenance [13, 14].

Up to now, collagen membrane is the one of favorable barrier materials used in GTR operation, as removal surgery and additional pain or infection are associated with the other non-resorbable membranes [14, 15]. However, the reconstruction outcomes are significantly depended on the stability of collagen membrane and regeneration space [16–18]. Hence, the barrier membrane is frequently used combined with bone grafts, as such bone substitute can maintain the space in bone defects as scaffolds for periodontium regeneration [19]. Unfortunately, the current regeneration approaches requiring clinical skill and experience are burdened by a significant amount of incomplete success. On the one hand, in treatment of defects with a non-supporting anatomy (i.e. wide defects with missing bony walls), the conventional granule-type bone grafts should be piled up to fit the defects [20]. And the compromised mechanical strength of bone substitute in unfavorable defect morphology may influence the wound stability and the regeneration space creation [21]. On the other hand, the barrier membrane needs to be tailored based on the defect for purpose of covering the defect completely. After shaping, the barrier should be placed between the gingiva flap and bone graft. The complication of anatomically alveolar contours makes trimming and placing resorbable membranes a time-consuming and critical procedure in regeneration surgery [17, 22]. In addition, whether the membranes matching the defect sites accurately and its long-term stability both remain uncertain.

Previously, 3D printing has been used to create custom implants in GTR treatment, leading to reduced surgery time, higher accuracy, and improved medical effects. In 2015, Rasperini et al. implanted a 3D printed polycaprolactone (PCL) scaffold containing recombinant human platelet-derived growth factor-BB (rhPDGF-BB) in a patient’s one wall bony defect [23]. Though the article reported an attachment gain of 3 mm, implanted scaffold was exposed intraorally at 13 months and removed. More recently, a biodegradable 3D woven-fabric scaffold containing autologous bone marrow stem cells and platelet rich plasma was applied in intrabony defects of 10 patients. An average linear bone growth of 4.7 mm was showed by 36 months [24]. Most recently, we also used β-tircalcium phosphate (β-TCP) and nonstoichiometric wollastonite (nCSi) to developed a core–shell bioceramic scaffold in customized shapes focusing on alveolar bone regeneration [20].

**Scheme 1:**
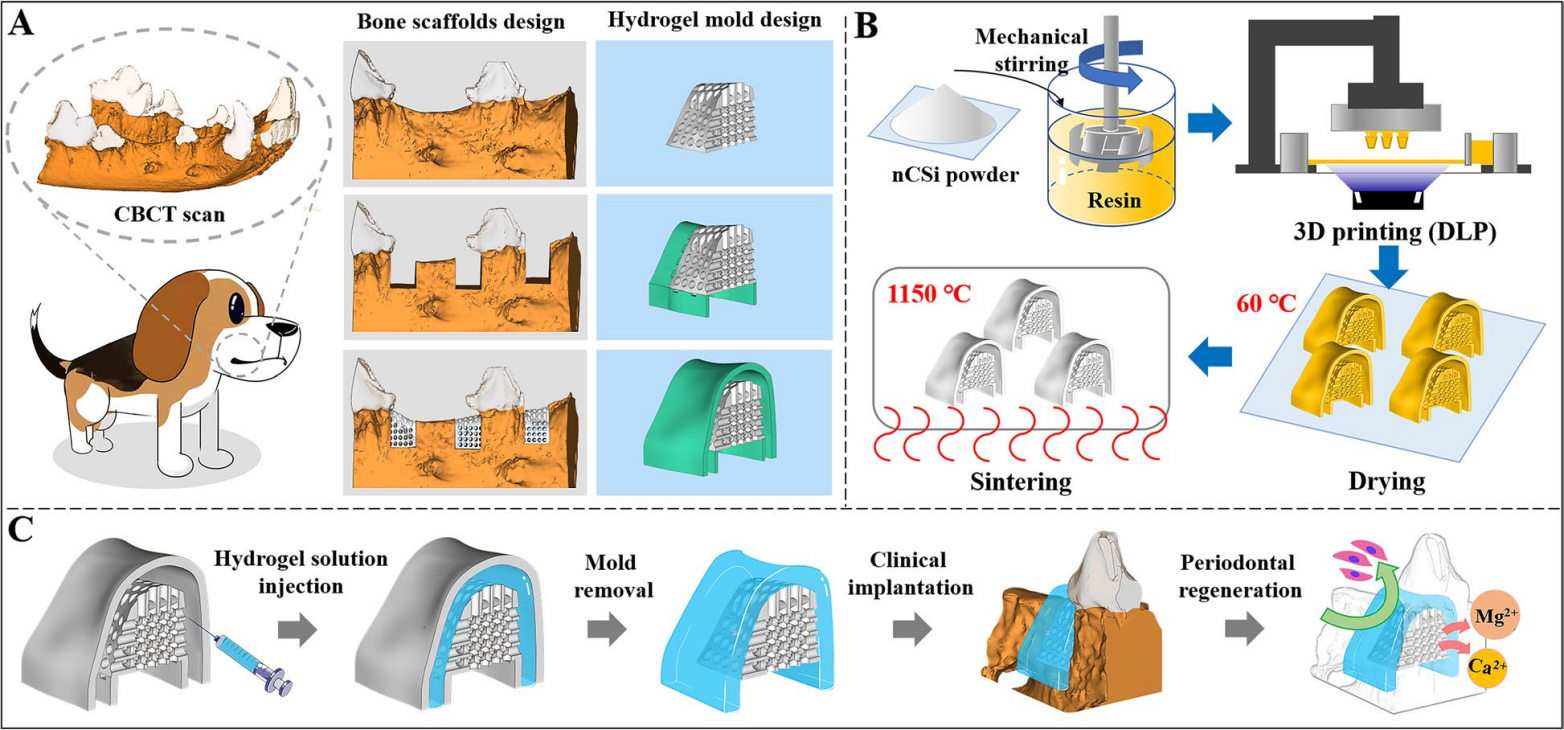
Schematic illustration for design and fabrication of organic–inorganic biphasic hierarchical architecture for guided tissue regeneration therapy

In the past decade, hydrogel-based biomaterials have been widely developed because of their self-adapting ability in variform periodontal defects. The injectable hydrogels containing chitosan or sodium alginate were prepared as a barrier membrane in GTR procedure [25, 26]. Whereas it is inevitable to wait for gelation reaction and membrane formation. Inspiring from the gelation mold strategy, we developed the hierarchical architectures composed of nCSi scaffolds doped with 6% Mg and gelatin methacrylate/silanized hydroxypropyl methylcellulose (GelMA/Si-HPMC) hydrogel membrane with personalized morphology to achieve the expected outcomes (see Scheme 1). The nCSi scaffolds were fabricated by digital light processing (DLP) to match the morphology of bone defects (Scheme 1A-B). The photo-crosslinked hydrogel injection technique was used to prepare the GelMA/Si-HPMC hydrogel in a specialized mold to fully integrate the morphology of bioceramic scaffolds and the periodontal defects (Scheme 1C). Si-HPMC is a self-setting hydrogel with a variety of biomedical applications, such as the barrier in periodontal regeneration to prevent cell invasion [26, 27]. In order to accelerate the crosslinking process of Si-HPMC, a mixture composed of Si-HPMC and GelMA was prepared because the latter is a gelatin-derived biomaterial with good photo-crosslinking ability [28–30]. Also, GelMA may tune an appropriate curing time for the mixture of GelMA/Si-HPMC and optimize the mechanical and biodegradable properties of the hydrogel membranes simultaneously.

Herein, we designed a biphasic hierarchical architecture containing nCSi scaffolds and GelMA/Si-HPMC membranes for resisting soft tissue ingrowth and guiding periodontium regeneration. The nCSi has been demonstrated as a new strength-strong bioactive ceramic beneficial for 3D printing high-porosity constructs [20, 31, 32]. Previous studies have shown that the degradation rate and mechanical strength of nCSi ceramics were apparently higher than that of common bioceramics such as β-TCP and hydroxyapatite (HA) [33, 34]. It is acknowledged that the slow degradation rate and high brittleness of ceramics can be disadvantageous for periodontal regeneration. Besides, the nCSi is easily fabricated as the 3D scaffolds and the bioactivity can be maintained after sintering [35]. Therefore, the personalized GTR strategy may enhance the periodontal regeneration by simplifying the surgery process and improving GTR predictability. The hierarchical architectures may accelerate the reconstruction of periodontium through preventing undesirable tissue invasion from root surface and further stimulating bone regeneration by ion dissolution products from bioceramic scaffolds and hydrogel membranes. The physicochemical properties and biological performances of the hydrogels with different ratio of GelMA and Si-HPMC were characterized in vitro systematically. Subsequently, a critical-size one-wall periodontal defect model in dogs was prepared to evaluate the periodontal tissue reconstruction potential of the biphasic hierarchical architecture.

## Materials and methods

### Materials

The reagent-grade calcium nitrate tetrahydrate (Ca(NO_3_)_2_·4H_2_O), magnesium nitrate hexahydrate (Mg(NO_3_)_2_·6H_2_O), sodium metasilicate nonahydrate (Na_2_SiO_3_·9H_2_O), and other inorganic reagents were purchased from Sinopharm Reagent Co., Ltd. The light-sensitive resin for DLP technique was supplied by Ten Dimensions Technology Co., China. GelMA (EFL-GM-90, DS: 90%) and photoinitiator lithium phenyl-2, 4, 6-trimethylbenzoylphosphinate (LAP) were purchased from Yongqinquan Intelligent Equipment Co., Ltd., Suzhou, China. The hydroxypropyl hethylcellulose (HPMC, METHOCEL™ E4M Premium CR) was provided by Shanghai Colorcon Coating Technology Ltd Shanghai, China. 3-glycidoxypropyltrimethoxysilane (GPTMS), n-Heptane and 1-Propanol were also bought from Sinopharm Reagent Co., Ltd. 4-(2-hydroxyethyl) piperazine-1-ethanesulfonic acid (HEPES) was bought from Sigma-Aldrich Co. LLC., USA.

### Synthesis of nCSi powders

The Mg-substituting-Ca nCSi (Ca_0.94_Mg_0.6_SiO_3_) powders were synthesized by wet-chemical co-precipitation procedure as described previously [36]. Briefly, Ca(NO_3_)_2_·4H_2_O, Mg(NO_3_)_2_·6H_2_O and Na_2_SiO_3_·9H_2_O were dissolved in the deionized water in a concentration of 0.5 mol/L separately, with 6% Ca(NO_3_)_2_ replaced by Mg(NO_3_)_2_. Then, the solution of Ca(NO_3_)_2_/Mg(NO_3_)_2_ was dropped into the Na_2_SiO_3_ solution under continuous magnetic stirring while the pH value was maintained between 9.0 and 10.0. After the reaction solution was aged for 12 h, the white precipitate was filtered, washed by deionized water for three times, and finally washed with ethanol for two times. The powder was dried at 80 ℃ for 24 h, and then calcined at 850℃ for 120 min. The chemical composition of the nCSi powders was measured by inductively coupled plasma-optical emission spectrometry (ICP-OES; Thermo, UK). And X-ray diffractometer (XRD; Rigaku, Tokyo, Japan) was used to verify the phase compositions of nCSi. The calcined bioceramic powders were ground by a planetary ball mill in ethanol medium for 6 h. The resulting particle size of the nCSi powders was below 5 μm.

### Preparation of Si-HPMC

Si-HPMC was prepared by grafting GPTMS to HPMC as described previously [37]. Briefly, 316 ml of n-Heptane were stirred with 70 ml of 1-Propanol in a ball glass, followed by adding 2.0 g of hydroxide sodium and 38.6 g of HPMC. The mixture was kept at room temperature for 45 min under a nitrogen bubbling. Then, 6 ml of GPTMS was added drop wise with a temperature increasing to 100℃ in 35 min. After 3 h of boiling and cooling till 40℃, 5 ml of acetic acid glacial was poured to neutralize the reaction. After 30 min, the mixture was filtered by a buschner and rinsed with 50 ml of acetone two times. The powder was dried at 50 ℃ for 1 h under vacuum. Finally, the dried powder was washed with 500 ml of a mixture acetone/water (85:15 v/v) three times and Si-HPMC powders were dried overnight at 50℃. FTIR studies were realized with a Spectrum Two FTIR (Perkin Elmer Inc., America), HPMC and Si-HPMC samples were at 1% in KBr. The silicon percentage grafted on HPMC (1.57% w/w) was determined by ICP-OES. Then Si-HPMC powder was dissolved in 0.1 M NaOH at 4% (w/v) overnight and sterilized by autoclave: 121℃ for 20 min. The acid buffer for Si-HPMC hydrogel was also prepared with 0.06 M HCl, 1.8% (w/v) NaCl and 6.2% (w/v) HEPES.

### Hydrogel preparation

To generate GelMA solution, GelMA was resuspended at 7.5% (w/v) in PBS with 0.05% (w/v) LAP and heated in a 60 °C water bath until it was completely dissolved. The GelMA solution was then incubated at 37 °C in a water bath, followed by sterilization using a 0.22-µm filter. The liquid formulation of Si-HPMC hydrogel was obtained by mixing 4% (w/v) Si-HPMC solution and acid buffer, previously described, in a 2:1 ratio in volume. The GelMA/Si-HPMC with different volume ratio of 6:0, 4:2, 3:3, 2:4 and 0:6 was respectively denoted as 6G0Si, 4G2Si, 3G3Si, 2G4Si and 0G6Si, and were poured into Teflon molds and irradiated 30 s under 405 nm curing light. Due to the overly slow condensation reaction, 0G6Si was kept in molds for 3 days to ensure fully crosslinking of the hydrogel. 6G0Si, 4G2Si, 3G3Si and 2G4Si were also preserved 3 days before tests to maintain the same conditions for all samples. The abbreviations of the hydrogel samples were shown in Table 1.

**Table 1:** The abbreviations of the hydrogel samples and implanted samples in beagle model

**Hydrogel sample**	**Volume ratio of GelMA and Si-HPMC**	**Abbreviation**
1	6:0	**6G0Si**
2	4:2	**4G2Si**
3	3:3	**3G3Si**
4	2:4	**2G4Si**
5	0:6	**0G6Si**
**Implanted sample**	**Detailed description**	**Abbreviation**
A	Bioceramic scaffolds	**nCSi**
B	Biphasic hierarchical architectures	**nCSi@3G3Si**

### Characterization of hydrogel

#### Scanning electron microscopy (SEM) analysis

To observe the fracture morphology of GelMA/Si-HPMC hydrogel, we examined the samples using a scanning electron microscope (SEM; S4800, HITACHI, Tokyo, Japan). Before the examination, the as-prepared composites (10 mm in diameter and 8 mm in height; *n* = 3) were freeze-dried. After that, the freeze-dried samples were sectioned to expose the inner structure, and then sputter-coated with platinum for SEM observation.

#### Rheology

Rheological measurements were taken on a rheometer (MCR302, Anton Paar, Austria). A light-curing system accessory was combined with an OmniCure® 365 nm light source (light intensity: 1 mW/cm^2^) allowing the material to be irradiated and start the gelation (*n* = 3).

#### Mechanical testing

All samples (10 mm in diameter and 8 mm high; *n* = 4) were incubated in PBS at room temperature for 12 h prior to testing. Mechanical compression was conducted using a mechanical tester (5543A, Instron, America) with a rate of 2 mm/min. The stress–strain curves were recorded and the compression modulus was calculated as the slope of the corresponding curve.

#### Swelling and degradation analysis

The mass of the hydrogel samples (10 mm in diameter and 8 mm high) was recorded before and after soaking in PBS at 37 °C. The dry samples of hydrogels (*n* = 4) were weighed (*w*_1_) first, then immersed in phosphate buffered solution (PBS) for 1, 2, 4, 8, 24, and 36 h, and respectively removed to be weighed (*w*_2_). The swelling ratio was calculated by the following formula:

1 $$\mathrm{swelling}\;\mathrm{ratio}=({\mathrm w}_2-{\mathrm w}_1)/{\mathrm w}_1$$

The mass remaining was measured by evaluated the dry samples (*n* = 3) weighted before and after being immersed in the PBS for 7, 14, 21, and 28 days, which were freeze-dried after removement.

### Cytotoxicity experiment

For cell proliferation, mouse fibroblast cell line L-929 cells (Cell Culture Center, Chinese Academy of Medical Science, China) were seeded respectively in a 96-well culture plate and cocultured with the impregnation solution of the hydrogels for 1, 3, and 5 days at 37 °C in a 5% CO_2_ atmosphere. The cells cultured with initial growth medium (Minimum Essential Medium α (MEM-α, Gibco, Thermo Fisher Scientific Inc., America.) supplemented with 10% fetal bovine serum (FBS, Gibco), 100 U/mL penicillin, and 100 μg/mL streptomycin) were chosen as control group. The cell proliferation was quantified by the CCK-8 assay (Yeasen Biotech Co., Ltd., China).

For live/dead cell staining, L-929 cells were seeded on the surface of hydrogels (5 mm in diameter and 2 mm in height) in a 24-well culture plate at a density of 3 × 10^3^ cells/well. After 4 and 7 days of culturing, the samples were stained with calcein-AM/PI (Beijing Solarbio Science & Technology Co., Ltd., Beijing, China). After incubation at 37 °C in the dark for 15 min, the inverted fluorescence microscope (Axio Observer 3, Zeiss, Germany) was used to observe the cells. living and dead cells were counted by ImageJ software.

### In vitro osteogenesis evaluation

Mandible-derived osteoblasts (MOBs) from bilateral mandibles of 1-day-old C57 mice were isolated and digested, as described previously [38]. The experimental protocols were approved by the Ethics Committee of the Second Affiliated Hospital School of Medicine of Zhejiang University (2021–130). In brief, the mandibles were removed, cut into small pieces, and washed with sterile PBS 3 times. Then, the bone pieces were placed in a T25 tissue culture dish to allow the cells grew out of the tissue-block. Subsequently, the cells were digested and cultured in Dulbecco’s modified Eagle’s medium (DMEM, Gibco) supplemented with 10% FBS, 100 U/mL penicillin, and 100 μg/mL streptomycin at 37 °C in a 5% CO_2_ atmosphere. Osteoblasts from passages two to five were used for the following experiments.

Cell scratch assay using osteoblasts were performed to evaluate the in vitro migration capacities of hydrogels. For the migration assessment, cells (1 × 10^5^ cells/well) were seeded in 6-well plates and incubated 24 h to reach a monolayered confluency. Linear wounds were scratched with a sterile 1 mL-pipette tip. Non-adhered cells were washed away by PBS. Then, the cells were incubated with 2 mL of different culture mediums with 1% FBS for 24 h. Four fields of the cells at 0 h and 24 h of incubation were randomly chosen, photographed, and migration areas were calculated based on ImageJ.

MOBs were seeded in 24-well plates (3 × 10^3^ cells/well) coculturing with the impregnation solution of hydrogels for 7 days. After 7 days coculture, cells were fixed with 4% paraformaldehyde solution (15 min), followed by PBS washing for 3 times, and stained with a BCIP/NBT Alkaline Phosphatase (ALP) Color Development Kit (Beyotime Biotechnology Co., Ltd., Shanghai, China). After incubation for 2 h in the dark at room temperature, the cells were imaged by the optical microscope (CKX53, OLYMPUS, Japan).

The osteogenic induction medium was prepared by the growth medium or impregnation solution containing 50 μg/mL l-ascorbic acid (Sigma-Aldrich Co. LLC., America), 10 nM dexamethasone (Sigma-Aldrich Co. LLC., America), and 10 mM β-glycerophosphate (Sigma-Aldrich Co. LLC., America). MOBs were seeded in 48-well plates (3 × 10^3^ cells/well) coculturing with different osteogenic induction mediums for 14 days. After 14 days of osteogenic induction, the formation of calcium deposits was evaluated by an Alizarin Red Staining (ARS) kit (Beyotime Biotechnology Co., Ltd., Shanghai, China). Images were captured with a digital camera and a light microscope (CKX53, OLYMPUS, Japan). Quantitative analysis was also conducted by adding 10% cetylpyridinium chloride in 10 mM sodium phosphate to destain the alizarin red (37 °C for 2 h). Then, the OD value was determined by absorbance measurement at 562 nm on a spectrophotometer.

### In vitro cell barrier effect analysis

The hydrogel samples with different GelMA/Si-HPMC ratio were prepared in Teflon molds (5 mm diameter and 2 mm in height). The hydrogels were preincubated in growth medium (DMEM supplemented with 10% FBS and 1% penicillin–streptomycin) in a 6-well plate for 24 h. After incubation, the medium was removed and the hydrogels were transferred to 24-well plates for cell seeding.

L-929 cells were seeded on the top of the hydrogels with 10 μL of cell suspension (3 × 10^3^ cells/hydrogel). After 1 h for cell adherence, growth medium was gently added to the bottom of the hydrogels and the samples were cultured for 7 days. The samples were then fixed by 4% paraformaldehyde and stained with Acridine Orange (AO), to visualize cell presence and morphology. The samples were then observed using confocal microscopy using 488-nm laser (LSM 900, Zeiss, Germany). Z-stacks from the top of the hydrogel surface to the thickness where no cells can be observed were obtained with a magnification of 100.

### Fabrication of biphasic hierarchical architectures

In order to obtain the region for one-well bone defects, the mandibular first and third premolars of beagle dogs were extracted bilaterally under anesthesia and healed for two months prior to the GTR surgery. To design the geometry and dimensions of the required architectures, the mandibula of the beagle dog was scanned with the Cone Beam computer tomography (CBCT) system (vivaCT100; Scanco Medical), as shown in Scheme 1. The CBCT scan images in the Digital Imaging and Communications in Medicine (DICOM) format were imported into the Mimics Research (19.0, Materialise, Belgium), a 3D image-based engineering software. Then the DICOM images were reconstructed into a 3D model, and exported in STL format. The above STL files were imported into Materialise Magics software (21.0, Materialise, Belgium), one-well intrabony defects (4 mm in mesiodistal width and 5 mm in depth) were designed on the distal and mesial of the second mandibular premolars and the mesial of forth mandibular premolars bilaterally. The customized scaffolds were designed according to the defects mentioned above with a porosity of 55% and an interconnecting pore dimension of 600 μm (Scheme 1A). Then hydrogel molds (SimpNeed, Niudai Technology Co., LTD, China) were assembled on the surface of bioceramic scaffolds which face the inside of gingiva, 1.5 mm was reserved for hydrogel membranes forming between the inner face of the molds and the bioceramic scaffolds (Scheme 1A). The outline of the molds was 2 mm beyond the margin of the defects or bioceramic scaffolds, which was similar to the conventional GTR membranes. Perforations were designed on the junction of the molds and bioceramic scaffolds for easily removing the molds subsequently. Finally, all 3D porous models of biphasic architectures were sliced to printing files and imported into the DLP 3D printer (Ten Dimensions Technology Co., China). The printing procedure depended on the photopolymerization of a mixture of resin and wollastonite powders (Scheme 1B). The resin and bioceramic powders were mechanical mixed in a mass ratio of 1:2. After stirring in a ball mill for 30 min, the slurry was poured into the slurry tank of the printer. Under an exposure time of 3 s for each layer with a light intensity of 10,000 μW/cm^2^, the slurry was solidified with a definite morphology and thickness, and stacked into the predetermined structure. After 3D printing process, the samples were ultrasonically washed in deionized water to clear the residual slurry followed by drying at 60℃ overnight. Subsequently, the porous samples were sintered in a muffle furnace (Kejing Co., Hefei, Anhui, China) at a target temperature of 1150℃ for 2 h (heating rate: 3℃/min) and cooled naturally. Then GelMA/Si-HPMC hydrogel solution were injected into hydrogel molds in two-phase architectures and irradiated 30 s under curing light for crosslink. After the hydrogel molds were removed by microsurgery forceps, customized inorganic–organic biphasic architectures (abbreviated as nCSi@3G3Si; Table 1) were completed (Scheme 1C). The finished architectures were sterilized by 10 MeV LINAC (CIAE-FZ-10/15, China Institute of Atomic Energy) with an irradiation dose of 15 kGy. The surface morphology and the bonding interface of organic–inorganic components were observed by SEM. The external and internal pore dimension of the bioceramic scaffolds after sintering were evaluated based on ImageJ.

### Periodontal tissue regeneration in beagle model

The one-wall intrabony defect model was used to evaluate the periodontal regeneration efficacy of biphasic architecture in vivo. The study was performed in accordance with the standard guidelines of Zhejiang University, experimental protocols were approved by the Zhejiang University Ethics Committee (ZJU20220055). Four male beagle dogs (10‒12 months old; 9.5 ± 1.2 kg in weight) were used. General anesthesia of dogs was administered by intramuscularly injection of Zoletil® 50 (1.0 ml/ body, Virbac S.A., France) following the local anesthesia by 2% lidocaine (1.0 ml/side, Shandong Hualu Pharmaceutical Incorporated Co., China). After teeth extraction and two months’ healing period, the dogs were anesthetized again, then the alveolar bone on the jaw bilaterally was exposed by elevating buccal and lingual mucoperiosteal flaps (Fig. 7Ai). Using a dental trephine bur, one-well intrabony defects were created (Fig. 7Ai) which were consistent with the previous design. Following root planning by Gracey curettes to remove the cementum and PDL on the root, a reference notch was also made into the root surface at the base of the defect. The two types of porous scaffolds with and without GelMA/Si-HPMC hydrogel membrane (nCSi, nCSi@3G3Si; Table 1) were then randomly implanted into the 16 defects, and 8 defect cavities without implantation were chosen as blank group (Fig. 7Aii). Then, the flaps were directly repositioned and sutured (Fig. 7Aiii). The animals were provided with a soft diet for 1 week, undergoing intramuscular injection with penicillin potassium (30,000 U/kg) for 3 days. After 4 and 8 weeks, the dogs were sacrificed respectively. The mandibles concerned were harvested for further analysis.

#### μCT measurement

After harvesting, the alveolar specimens were fixed in 4% formalin and measured by microcomputer tomography system (μCT; Inveon μCT scanner, Siemens, Germany). The specimens were scanned along the mesiodistal direction of mandible covering the entire region of intrabony defects under an exposure parameter of 80 kV and 80 mA. The region of interest (ROI) was selected at the periodontal intrabony defects and virtually 3D reconstructed, the newly formed bone (NB) volume/total volume (BV/TV) ratio and trabecular number (Tb. N) were calculated.

#### Histological analysis

After μCT measurement, the samples were rinsed with tap water overnight and dehydrated with ascending series of alcohol solutions from 70 to 100%, and then embedded in polymethylmethacrylate. The harden specimens were cut into slices with a thickness of 200‒300 μm along the mesiodistal direction of defects by a saw microtone (SP1600, Leica, Germany). Finally, the original slices were ground and polished to a final thickness of 40‒50 μm, and then stained with hematoxylin/eosin (HE) and MacNeal's trichrome staining. A light microscope (DMLA, Leica, Germany) was used to observe and record the stained sections under 20 × , 40 × and 100 × magnifications. Histomorphometric analyses were conducted by ImageJ, a semiautomatic image analysis software. The distance from the base of the defects to long junctional epithelium and the length of regeneration PDL in MacNeal's trichrome staining were calculated (*n* = 4).

### Statistical analysis

All statistical procedures were performed by SPSS 19.0 (SPSS, Chicago, IL, USA). Quantitative data were expressed in terms of mean value ± standard deviation (SD), Student’s test and one-way ANOVA test were conducted for statistical analysis. In all cases, *p* < 0.05 was regarded as statistically significant.

## Results

### Analysis of Si-HPMC and nCSi powders

**Fig. 1:**
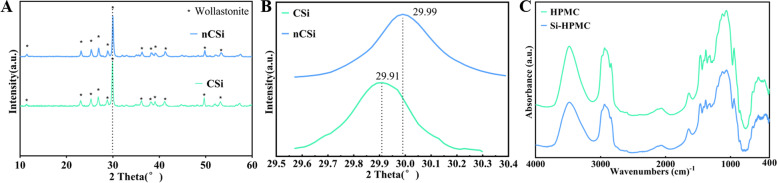
XRD patterns of the wollastonite bioceramics without and with Mg doping (**A**, **B**) and FTIR spectra of Si-HPMC (**C**) powders. The single peaks in (**B**) are the magnification of (-2, -2, 2) crystal plane diffraction peak

The ICP analysis indicated that the silicon percentage grafted on HPMC was 1.57% (w/w) and the Mg‐substituting‐Ca ratio in nCSi was 6.67 mol%. The XRD pattern (Fig. 1A) confirmed that the characteristic peaks of nCSi powder were consistent with the wollastonite phase. Because the Ca^2+^ (1.00 Å) in the wollastonite lattice was partially replaced by Mg^2+^ (0.66 Å), a slight peak shift of (-2, -2, 2) crystal plane to higher 2*θ* value (from 29.91° to 29.99°/2*θ*) can be observed (Fig. 1B). No difference was observed between silanized-HPMC and HPMC by FTIR analyses (Fig. 1C), indicating that the chemical modification was not strong enough to be detected.

### Characterization of hydrogel

**Fig. 2:**
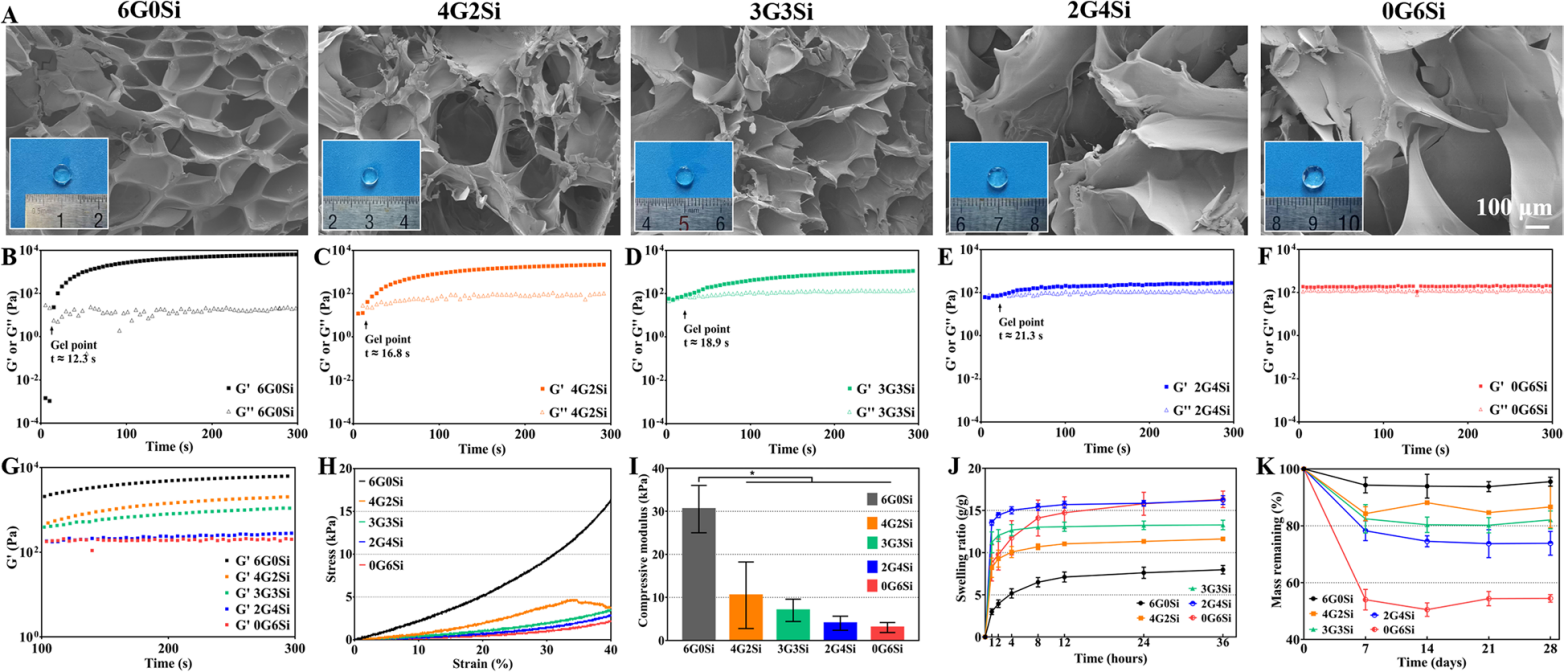
Primary physicochemical and mechanical characterizations of the GelMA/Si-HPMC hydrogels different mass ratio of GelMA (G) and Si-HPMC (Si). **A** Outward appearance and SEM observation of the hydrogels; **B**-**G** Rheological analysis involving the storage modulus (G′) and loss modulus (G′′) under 1 mW/cm^2^ 365 nm light irradiation; **H** Representative compressive stress–strain curves of the hydrogels; **I** Compressive strength of the hydrogels; **J** Swelling curves of the hydrogels; **K** Mass decay of the hydrogels during immersion in PBS at 37℃. * *p* < 0.05

The typical 3D porous architectures of as-dried GelMA/Si-HPMC hydrogels were evaluated by SEM observation (Fig. 2A). It can be seen that the 6G0Si and 0G6Si exhibited similar porous structures but different pore size in the fracture surface layer. The 6G0Si showed obvious lower micropore dimension than those in the 0G6Si sample. While the 4G2Si, 3G3Si and 2G4Si samples showed a combination of structure which gradually transformed from GelMA to Si-HPMC. The outward appearance of hydrogels was also observed by a digital camera (Fig. 2A, inset). It could be seen that all hydrogels retained the defined shape determined by the Teflon molds after crosslinking, without remarkable shrinkage or deformation. In accordance with the SEM observation, the swelling ratio of 6G0Si, 4G2Si and 3G3Si hydrogels were lower than those of the 2G4Si and 0G6Si samples (Fig. 2J), implying that the network structure of 4G2Si and 3G3Si hydrogels was stronger and more stable compared with 0G6Si.

Time-sweep experiments were performed to analyze the gel point and monitor changes in G′ and G′′ of different hydrogels under UV irradiation (Fig. 2B-G). The gelation time of the GelMA-containing hydrogels were less than 30 s, which superior to the self-setting 0G6Si hydrogel, and displayed higher elastic modulus. As for the high-GelMA samples (i.e. 6G0Si and 4G2Si), a sharp and immediate increase of elastic modulus can be observed, while the 3G3Si and 2G4Si samples showed a gradual increase. Due to the slow crosslinking by silanol condensation, the elastic moduli for the 0G6Si sample stayed constant in 300 s. The initial higher G′ compared with G′′ in 0G6Si suggests the existence of physical interactions between polymer chains. It is obvious that hydrogels with a higher ratio of GelMA can form hydrogels with higher elastic modulus or otherwise better elasticity and strength (Fig. 2G).

The mechanical analysis of hydrogels (Fig. 2H, I) showed that, the compressive strengths for the mixtures (4G2Si, 3G3Si, 2G4Si) were enhanced with the increase of GelMA, though there were no significant difference in comparison with the 0G6Si samples. The decreasing stress at 30 to 40% strain in stress–strain curve of 4G2Si hydrogel (Fig. 2H) indicated that the corresponding hydrogel sample was crushed under the increasing of strain. This phenomenon is common in mechanical compression test. Figure 2K showed the mass decay of hydrogel samples during immersion in PBS for 28 days, which may reflect the stability in biological environment. 6G0Si and 0G6Si samples showed the slowest and fastest mass decay, respectively. As for the composite hydrogels, the mass was decreased with increase of GelMA content, and the mass residual of biphasic hybrids was significantly higher than that of 0G6Si.

### Cytocompatible analysis

**Fig. 3:**
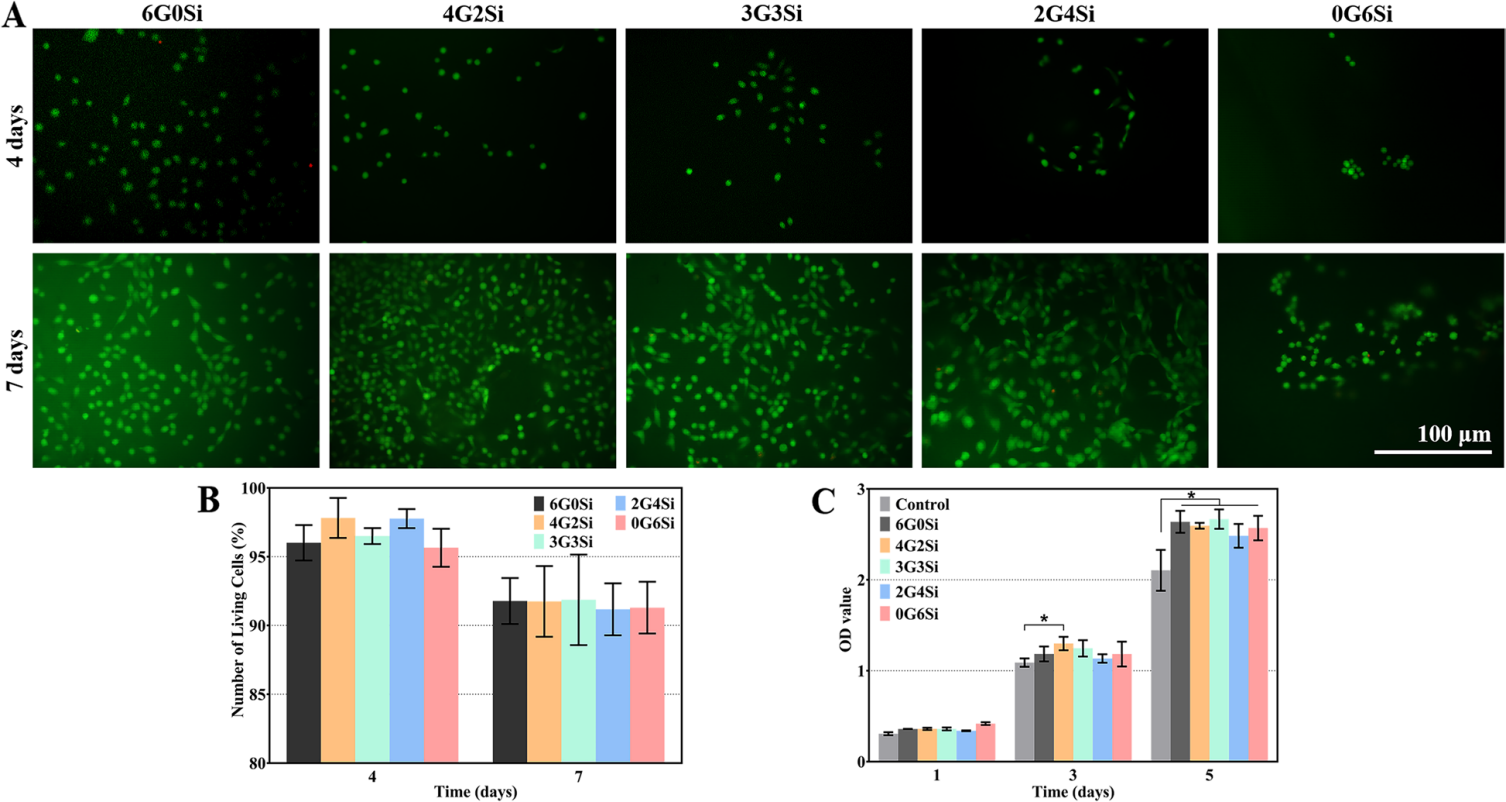
Live/dead fluorescence (**A**) and quantitative analysis (**B**) of L-929 cells cultured in the surface layer of GelMA/Si-HPMC hydrogels for 4 and 7 days, respectively, and CCK-8 assay (**C**) of L-929 cell culturing for 1‒5 days. * *p* < 0.05

L-929 cells were employed to evaluate the cellular compatibility of the GelMA/Si-HPMC samples by the live/dead cell staining experiments. After staining, green fluorescence would be emitted by the living cells under the action of calcein-AM, while PI would enter dead cells and generate red fluorescence (Fig. 3A). After 4 days, L-929 cell adhesion was observed on the hydrogels. Better cell migration and spreading were identified on the GelMA-containing hydrogel samples. The cells were further increased after 7 days, and showed a typical round or spindle morphology on the surface of samples. More cells exhibited spindle morphology on GelMA-present hydrogels, while the cells on Si-HPMC sample (0G6Si) were more agminated. Even so, all the hydrogels showed good cytocompatibility as only a few dead cells can be observed. Quantitative analysis of the number of living cells was consistent with the microscope observation (Fig. 3B). In order to quantify the proliferation of cells, cell counting using CCK-8 was performed (Fig. 3C). On day 1, no significant difference in OD value was found among six groups. At 3 days, the OD values of the 4G2Si group were statistically higher than that of the control group (*p* < 0.05). Then at 5 days, all the groups displayed higher OD value than control group and the differences were statistically significant (*p* < 0.05). The above results demonstrated that the cells contacting with hydrogels extract or hydrogels did not exhibit a decrease in cell viability.

### Migration and osteogenesis of osteoblasts

**Fig. 4:**
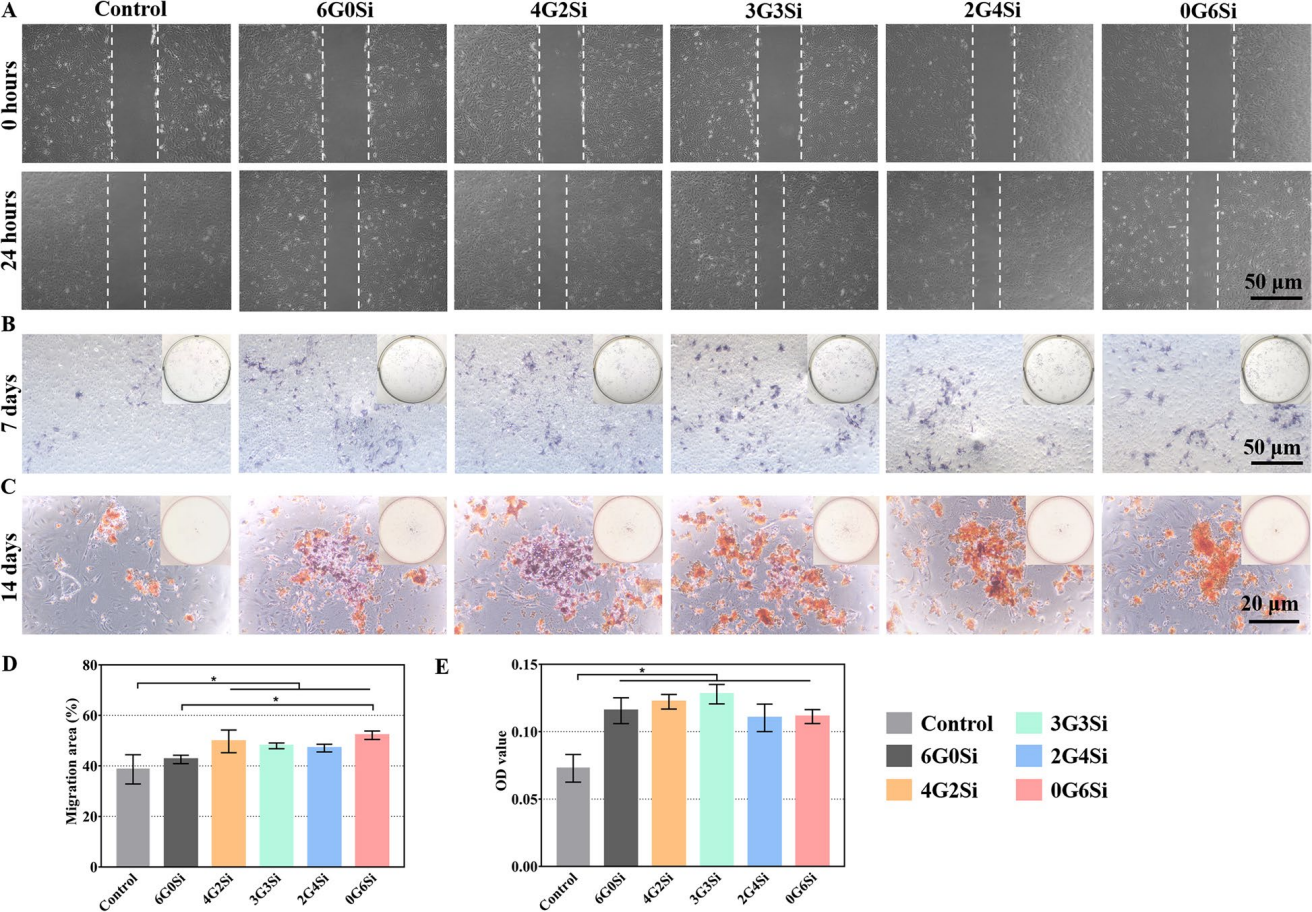
**A** Scratch assay of MOBs cultured with the impregnation solution of hydrogels for 0 and 24 h; **B** ALP staining of cultured MOBs after 7 days; **C** alizarin red staining (ARS) and quantitative analysis **E** of MOBs cells cultured with impregnation solution of hydrogels after 14 days; **D** quantitative analysis of migration area of cells. * *p* < 0.05

The wound healing efficiency of the hydrogel membranes was compared with the control growth medium. According to the microscopic images obtaining at 0 and 24 h, the scratch area is drastically reduced in the Si-HPMC-containing hydrogel-treated wells compared to the control group (Fig. 4A). Furthermore, quantitative analysis verified that the migration areas of MOBs in these hydrogels were significantly bigger than control group, and the wound closure was better in the 0G6Si group when comparing with the 6G0Si group (Fig. 4D). This scratch assay suggested that the Si-HPMC-containing composite hydrogels may improve periodontal healing efficiency by promoting osteoblast cell migration.

To assess the osteogenic activity of hydrogel materials, ALP and AR staining were examined. At 7 days, ALP staining showed that its expression in MOBs cultured with GelMA/Si-HPMC hydrogels was significantly higher than that of the control groups (Fig. 4B). Similarly, the AR staining for qualitative and quantitative analyses showed remarkably increased calcium deposition in the hydrogel membrane groups on day 14 (Fig. 4C, E).

### In vitro barrier effect

**Fig. 5:**
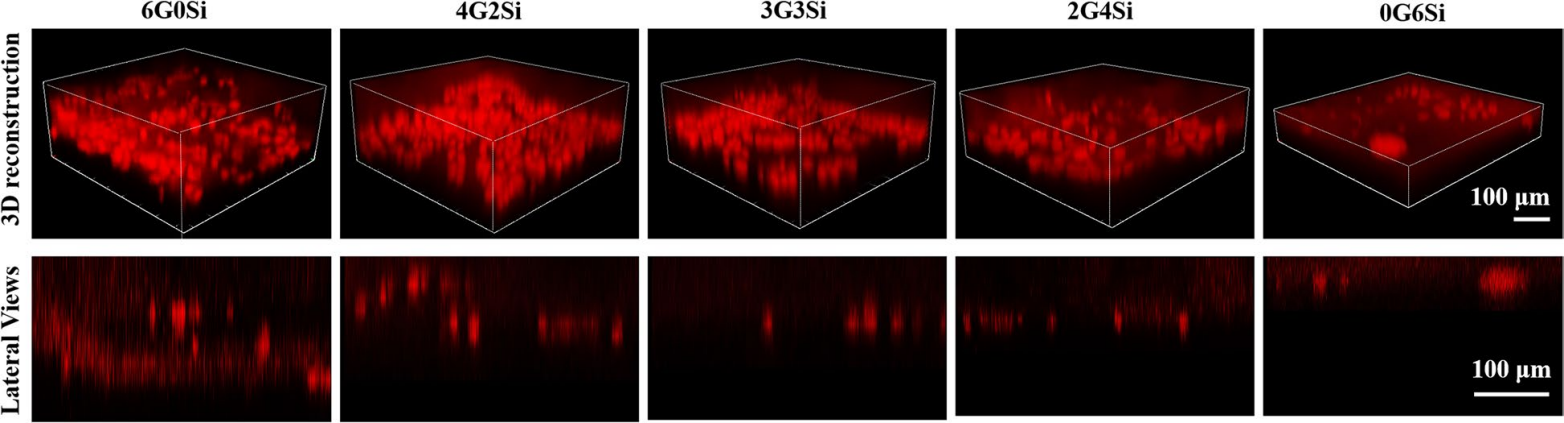
Hydrogel barrier effect against L-929 cells. Confocal microscopy images of cells seeded on the surface of hydrogels after 7 days. Top: 3D reconstruction of volume observed from hydrogel top surface to a thickness where no cells can be observed. Bottom: lateral views of cells on the surface of hydrogel membranes

The basic functional requirement of the membrane unit used for GTR is the cell barrier effect. To prove the cell obstruction, L-929 cells were seeded on the top surface of hydrogels for 7 days. Figure 5 showed the 3D reconstructions and lateral-view 2D projections for the cells. It was interesting that almost all the cells could be observed on the top of the membrane samples. As for the Si-HPMC hydrogel (0G6Si), the cells could organize to a cluster and the barrier efficacy was clearly confirmed by lateral projection view. However, more and thicker cell layers can be observed on the top of GelMA (6G0Si) and 4G2Si samples. According to the lateral view, a few cells have infiltrated in the 6G0Si and 4G2Si membranes. In the 3G3Si and 2G4Si groups, few infiltrating cells can be found form the lateral projection. The 3D reconstruction also showed thinner cell layers in comparison with the 6G0Si and 4G2Si groups. Although the cell distribution was more extended than 0G6Si hydrogel, the occlusive aspect of 3G3Si and 2G4Si samples was confirmed. Therefore, the 3G3Si hydrogel with expected physiochemical properties and biological activity was chosen for the following studies.

### Structural characterization of hierarchical architectures

**Fig. 6:**
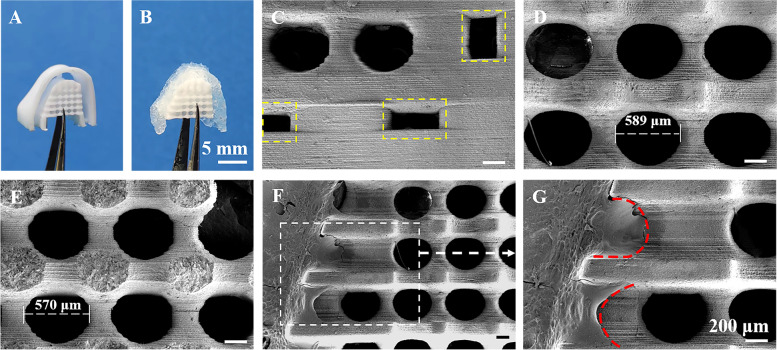
Primary morphology and structure observation for the biphasic hierarchical architectures. Outward appearance before (**A**) and after (**B**) combination of hydrogel membrane; **C** SEM image of the perforations on the connection area of hydrogel mold and porous nCSi scaffold (in yellow dotted box); SEM images of the outer surface (**D)** and fracture surface (**E**); **F** Interface region between the hydrogel membrane and bioceramic scaffold at low magnification. Scale bar: 200 μm. The white dotted box and arrow indicate the microstructure of interface (**G**); Red dotted lines indicate the infiltration of hydrogel in the porous structure of scaffolds

According to the procedure mentioned before, the biphasic hierarchical architectures containing nCSi scaffolds and 3G3Si membranes (nCSi@3G3Si) were fabricated successfully (Fig. 6A, B). The SEM observation indicated that perforations were add on the connection area of hydrogel mold and porous nCSi scaffold for removing the mold easily (Fig. 6C). The pore dimension (external, 592.6 ± 8.8 μm; internal, 562.3 ± 10.4 μm) was similar to the designed value (600 μm), and the pore size from the vertical printing direction was slightly decreased after sintering (Fig. 6D-E). These flaws can be further optimized by adjusting the printing models of architectures because of the high fidelity and precision of the DLP process. The organic–inorganic bonding interface was also observed by SEM (Fig. 6F-G). The high magnification image exhibited that hydrogel membrane integrating tightly with the bioceramic scaffold. Besides, the hydrogel infiltrated in the macropore structures on the surface layer of macroporous scaffold without blocking the internal pore structure.

### Animal surgery and μCT reconstruction analysis

**Fig. 7:**
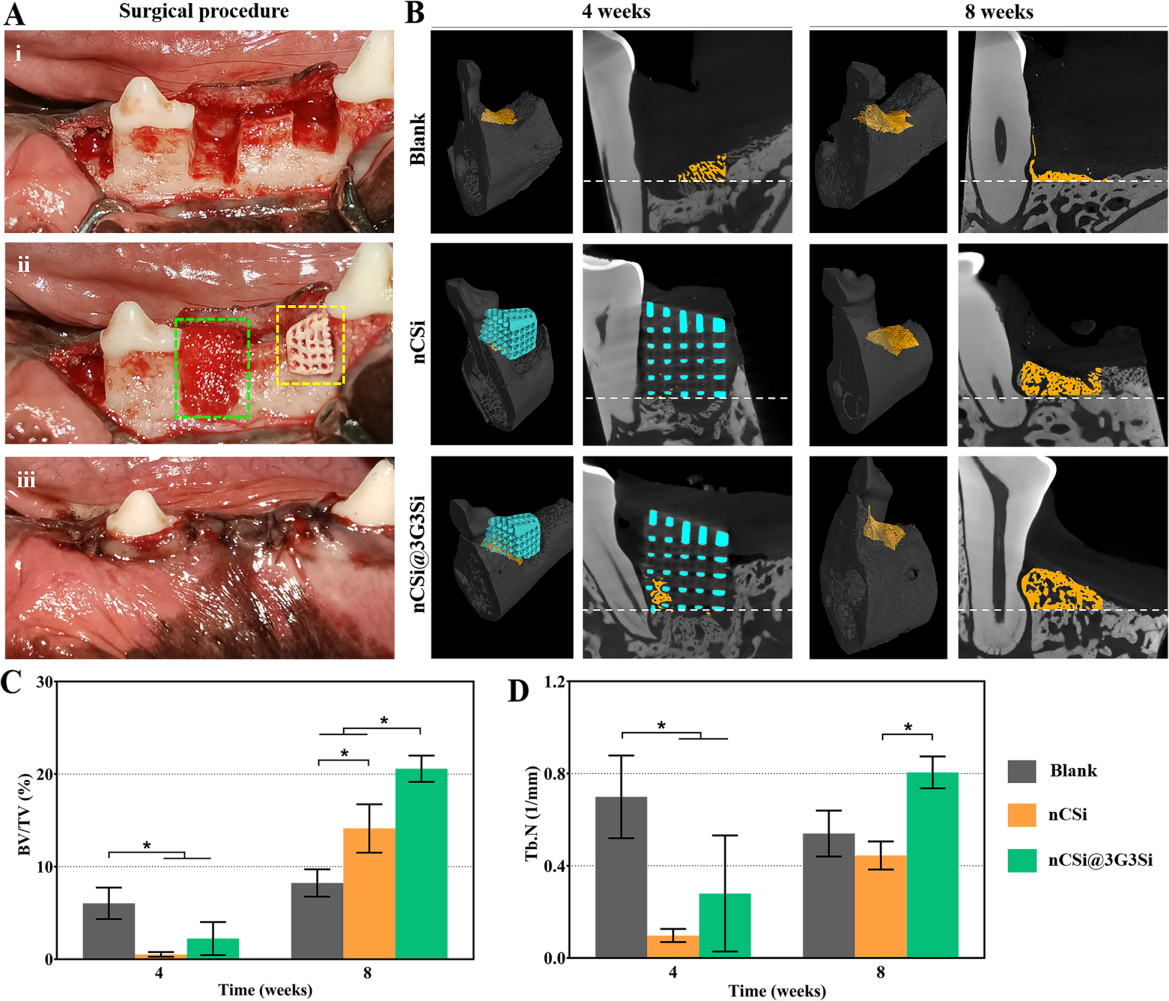
Surgical process and reconstructed analysis of the bone specimens. **A** Surgical procedure of one-step GTR approach; (i) Mucoperiosteal flap elevated to expose the alveolar bone and defects creation; (ii) nCSi (yellow dotted box) and nCSi@3G3Si (green dotted box) implantation; (iii) Flap was sutured in position. **B** 2D/3D μCT reconstructed images of the one-wall intrabony defects filled with nCSi and nCSi@3G3Si at 4–8 weeks postoperatively (Dotted lines indicating the bottom of the defects. Yellow: new bone; blue: material residual). **C**, **D** Quantitative data of the newly formed bone volume/total volume (BV/TV) ratio and trabecular number (Tb. N) based on μCT (* *p* < 0.05, *n* = 3)

The porous scaffolds without and with integrating hydrogel membrane (nCSi, nCSi@3G3Si) were implanted into one-wall intrabony defects to evaluate the periodontal regeneration efficacy in vivo (Fig. 7A). The scaffold-based porous architectures were implanted in the defects directly, without bone grafts shaping, barrier membrane tailoring or fixation. It is evident that the technical sensitivity of the GTR surgery is reduced due to personalized porous architectures. According to the μCT analyses (Fig. 7B), minor new bone tissue could be observed in the bone defects in blank group at 4 weeks, but after 8 weeks the height of alveolar bone was obviously decreased. In nCSi and nCSi@3G3Si groups, distinct structures of scaffold can be seen in the defect cavities at 4 weeks with new bone tissue ingrowth into the macropores of scaffolds. Bioceramic scaffolds played an important part in maintaining the height of alveolar and were replaced by newly formed bone after 8 weeks. It was obvious that more bone tissues can be observed in nCSi and nCSi@3G3Si groups compared with blank group at 8 weeks, when the bone volume was the highest in the hierarchical porous architectures (nCSi@3G3Si group). Additionally, the structure of new bone became more mature in all the groups during 4–8 weeks period.

On the other hand, the quantitative analyses of BV/TV and Tb. N were consistent with the qualitative 2D/3D reconstruction observation (Fig. 7C-D). According to the IAW analysis, significantly higher BV/TV and Tb. N data in the nCSi@3G3Si group implied an appreciable alveolar bone ingrowth at 8 weeks postoperatively. Meanwhile, significantly lower value of BV/TV and Tb. N in the nCSi group comparing with the nCSi@3G3Si group at 8 weeks indicated the limitation of new bone tissue regeneration due to absence of hydrogel membrane unit. Also, the nCSi group showed higher value of BV/TV in comparison with the blank group at 8 weeks (*p* < 0.05), implying the bioceramic scaffolds alone do have a certain effect on osteostimulating effect in the alveolar defects.

### Histological and histomorphometric analysis

**Fig. 8:**
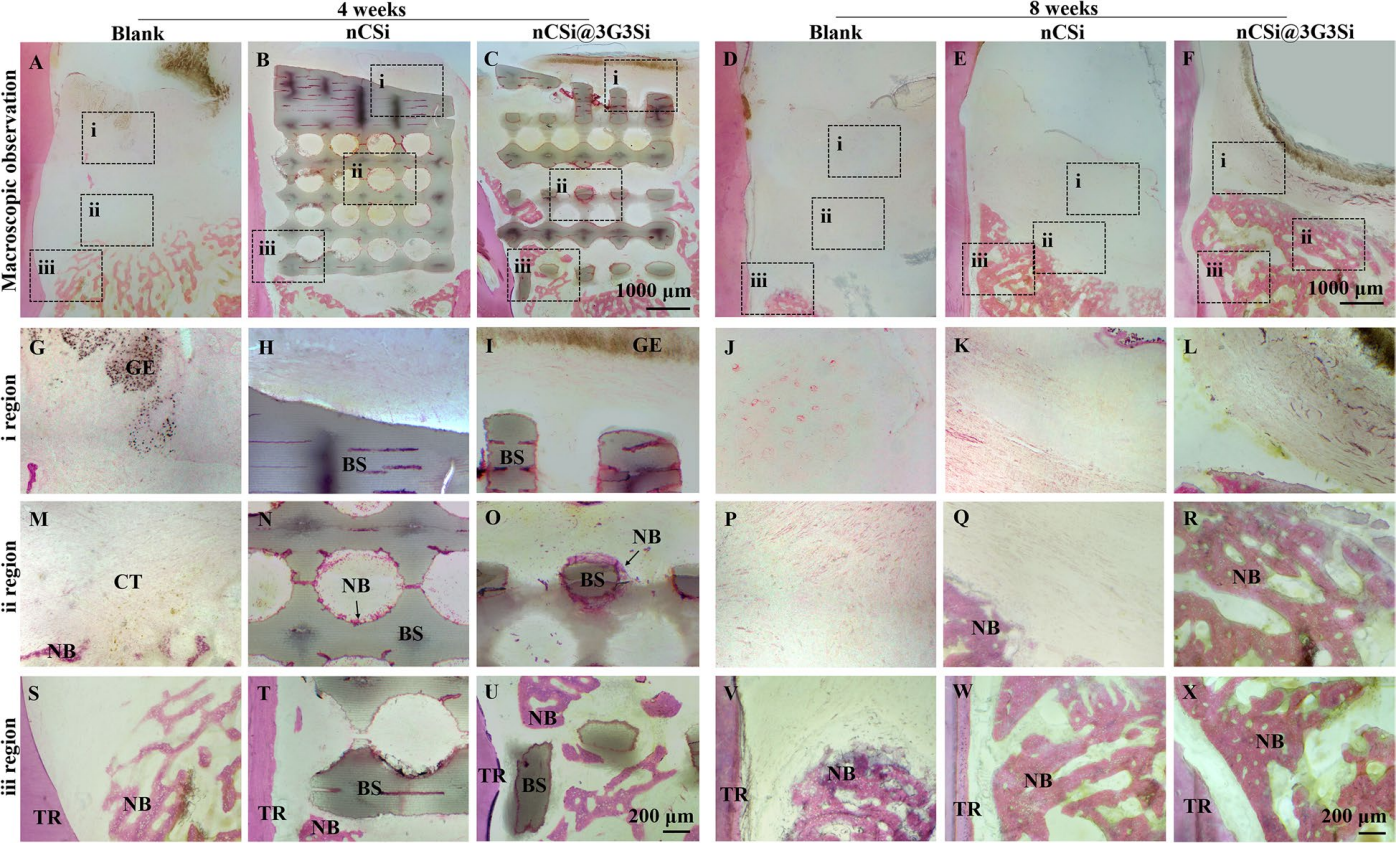
**A**-**F** H&E staining of new bone formation in defects at 4–8 weeks postoperatively (*n* = 3). **G**–**X** show i, ii, and iii regions of the defects (TR: tooth root, NB: newly formed bone, BS: bioceramic scaffold, GE: gingival epithelium, CT: connective tissue)

Histological staining analysis was conducted to explore the periodontal tissue healing in one-wall intrabony defects. Figure 8 shows the new bone grew in the porous networks of the bioceramic scaffolds (Fig. 8N) or surrounded the structure of the scaffold (Fig. 8O) after 4 weeks. Though appreciable new bone tissue was observed at the base of the defect in the blank group (Fig. 8S), the alveolar defects was mainly occupied by the gingival epithelium (Fig. 8G) and connective tissue (Fig. 8M). Meanwhile, nCSi scaffolds can be observed in the center of the defects (Fig. 8N-O) and under gingival epithelium (Fig. 8H-I) which still played an important part in maintaining the regeneration space in the alveolar defects. No traces of the hydrogel membrane were found between the gingival epithelium and the surface of scaffolds. Then, nCSi and nCSi@3G3Si were fully biodegraded at 8 weeks and successfully replaced by woven bone tissue. As for the blank group, only a little matured bone tissue can be seen and the alveolar bone was decreased obviously. Meanwhile, more new bone formation was showed in the nCSi@3G3Si group compared with nCSi group, suggesting the hierarchical porous architectures may be biodegraded over time, accompanying with launching new bone ingrowth efficiently.

**Fig. 9:**
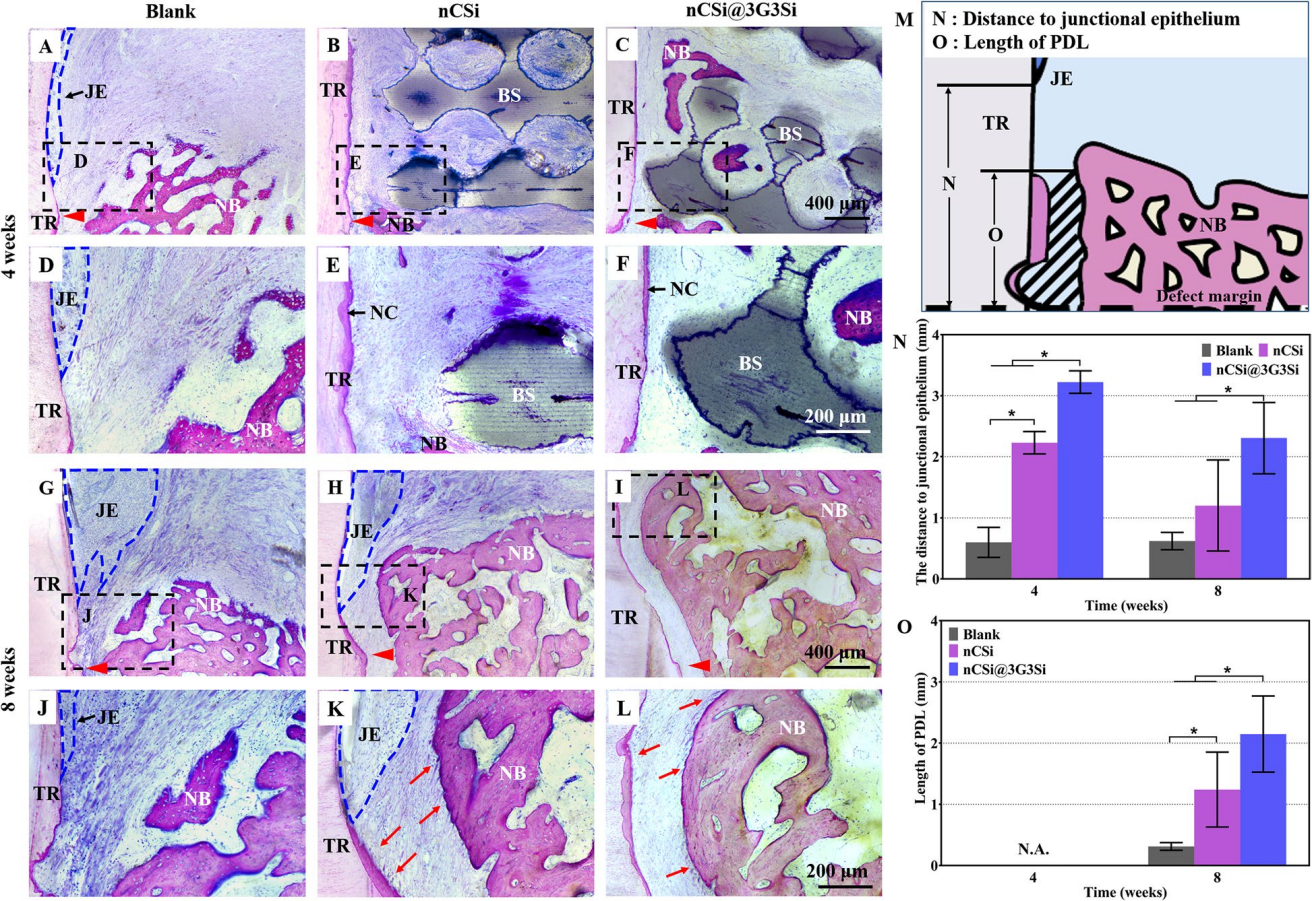
MacNeal's trichrome staining of periodontal tissue regeneration at the base of the defects at 4–8 weeks postoperatively. **D**–**F** and **J**-**L** show magnified structure in the base of the defects with tooth root stained in pink, the newly formed bone or cementum dyed in red purple, the junctional epithelium dyed in blue, and the connective tissue stained in lavender (TR: tooth root, NB: newly formed bone, NC: newly formed cementum, BS: bioceramic scaffold, JE: junctional epithelium). Red triangle: the base of the defects; the dashed blue area: ingrowth of junctional epithelium; red arrows: periodontal ligament (PDL). **M** Schematic illustration of the distance between the base of defect to junctional epithelium (**N**) and the length of PDL (**O**)

MacNeal's trichrome staining was used to evaluate the maturation of bone, regeneration of PDL as well as the migration of junctional epithelium. As shown in Fig. 9, the newly formed bone or cementum was dyed in red purple, when the junctional epithelium was dyed in blue. Evidently, a few new bone tissues can be seen in the blank group with long junctional epithelium approaching the base of the defect for 4 weeks postoperatively (Fig. 9A, D, the dashed blue area). Though the new bone formation was not as expected in the defects filled with the nCSi or nCSi@3G3Si, no distinct structure of epithelium was found on the surface of root (Fig. 9E, F). Evidently, newly formed cementum can be observed on the root. After 8 weeks of implantation, PDL anchoring between root cementum and alveolar bone was formed in the nCSi@3G3Si and nCSi groups (Fig. 9K-L, red arrows). However, long junctional epithelium still migrated close to the bottom of the defects in the nCSi group. The PDL regeneration in the blank group is very limited because of the inadequate formation of alveolar bone and cementum (Fig. 9G, J). Quantitative analysis (Fig. 9O) showed significantly longer PDL in the nCSi@3G3Si group after 8 weeks, indicating more efficient periodontal regeneration. The significant long distance from the base of the defects to junctional epithelium revealed the barrier effect of 3G3Si in the hierarchical porous architectures (Fig. 9N).

## Discussion

The optimal requirements to design a multifunctional scaffold for periodontal defects include bioactive, biodegradable, structural supporting properties and clinical manageability. However, few studies have evaluated strategies to reduce the operation sensitivity in GTR surgery; therefore, novel therapeutic approaches are needed to improve the predictability of periodontal regeneration. In the current study, we have developed a biphasic hierarchical architecture designed based on the architectural arrangement of alveolar bone, which provides a bioactive core scaffold for bone ingrowth and an external hydrogel membrane layer for resisting soft tissue ingrowth. Basically, the porous hydrogels can facilitate transportation of nutrition, oxygen, waste as well as the infiltration of blood vessels [39], which is crucial in periodontal regeneration process. Additionally, pore size is closely related to both the cell occlusive property and PDL/bone regeneration. Previous studies have demonstrated that macroporous design (pore size > 100 μm) is favorable to provide adequate space for osteogenesis cell ingrowth, which leads to greater bone regeneration compared to microporous membrane [40, 41]. However, as for barrier functionality, it has been reported that membranes with pores size exceeding 100 μm may permit soft‐tissue invasion [42]. It is obvious that the invasion of soft‐tissue cells may recolonize the defect site and inhibit the infiltration and activity of PDL‐forming cells. In this aspect, if the permeability required for periodontal tissue regeneration is fulfilled, the cell occlusivity will in turn be negatively affected. Actually, the optimal pore size of membrane, ranging from solid to macroporous, varies between the available membranes, and the optimal membrane pore size has probably not been defined to date. [43, 44] Although the specific effect of pore parameters is not involved in the present work, the porous architectures of hydrogels can be simply tuned by adjusting the concentration of polymeric hydrogel [45]. Meanwhile, the hydrogel membrane also exhibited adjustable mechanical, biodegradable properties and favorable biological performances including osteogenesis capacity and barrier effect, because it presents a synergistic combination of the favorable capabilities of GelMA and Si-HPMC.

In general, the mechanical properties of barrier membranes should meet the clinical need to maintain structural integrity in surgery, as well as withstand forces from regenerating tissue and oral activities after surgery [46, 47]. Our organic–inorganic (biphasic) hierarchical porous architectures solve the problem that the membranes absence of expected mechanical property usually fail to repair the periodontal tissue efficiently. Indeed, the mechanical strength of the hydrogels was improved slightly (Fig. 2H-I), and meanwhile the integration with strength-strong nCSi scaffolds can avoid the structural collapse in the defects for a long-time stage. In addition, the hydrogel membrane in the biphasic porous architectures is finely designed, and tailoring or fixing the membrane is not needed in the surgery.

On the other hand, the barrier membrane should be maintained intactly during the early time stage of wound healing to prevent the migration of epithelial cells [22]. Accordingly, it is extremely important to control the degradation rate of GelMA/Si-HPMC hydrogels. Si-HPMC has been reported to show at least 8 weeks’ degradation in a rabbit femoral epiphysis defect model [48]. Our results indicate that the 3G3Si hydrogel is more sparingly dissolved in PBS than the Si-HPMC (0G6Si), implying the GelMA‒Si-HPMC system may prolong the barrier effect in periodontal defects (Fig. 2K). Moreover, it is well agreed that the barrier biomaterials should be biocompatible with the endogenous cells to support tissue healing process [40]. GelMA and Si-HPMC are both biodegradable and injectable hydrogel that is nontoxic, and have been widely studied for tissue engineering or drug delivery applications [49, 50]. The cytocompatibility of hydrogels was evaluated in our study to confirm the nontoxic effect of the residual chemical reagents in producing process. Live-Dead staining analysis results indicate that the cells can spread well on the surface of the GelMA-containing hydrogels (Fig. 3A). Because GelMA can facilitate cell adhesion and spreading due to the RGD sequence which possesses high affinity for the integrin receptors on the cell membrane [51]. In contrast, the Si-HPMC hydrogel (0G6Si) can maintain the cell in a non-adherent shape and form a protective barrier [52]. Thus, it is reasonable to assume that the improved initial contact of cells on the composite hydrogels might attribute to the RGD sequence from GelMA.

In addition, ideal periodontal membranes are expected to favor the osseointegration with host tissues aiming for better periodontal regeneration. Our composite hydrogels with considerable osteogenesis capacity may give the expected biological performances according to the ALP and AR staining analysis (Fig. 4). On the one hand, it has been reported that GelMA can promote ALP expression and ECM mineralization during osteogenesis [53, 54]. And the incorporation of Si from Si-HPMC is also essential for such metabolic processes, possibly leading to the improvement of osteogenic activity [55, 56]. On the other hand, the periodontal defect repair relies on the migration, differentiation, and extracellular matrix production of host osteogenic cells [57]. As shown in cell scratch assay (Fig. 4A), it was clear that the groups containing Si-HPMC showed significant improvement in cell migration. It is very likely that the positive effect on MOBs migration is attributed to the silicon release during the hydrogel degradation. Previous studies also proved that silicon-based treatments or biomaterials may enhance cell migration and wound healing [58–60]. In general, such composite membranes probably exhibit a synergistic effect on promoting the migration and differentiation of osteoblasts, and thus can be a promising candidate for promoting periodontal bone regeneration.

It is worth mentioning that the cell barrier effect is the most important characteristic of GTR membrane. In this study, the potential barrier effect of hydrogel membrane was assessed systematically via cell experiments. After 7 days’ co-culture, L-929 cells were found on the surface of the membranes (Fig. 5). The cells appeared more elongated in the groups containing GelMA, and started to infiltrate the hydrogel in 6G0Si and 4G2Si groups. This could be due to more interactions between the cells and the hydrogels. In contrast, a higher content of Si-HPMC may alleviate these interactions. In the periodontal one-wall intrabony defect model, the occlusive aspect of the hydrogel membranes was then analyzed by MacNeal staining (Fig. 9). The much longer distance from the base of the defects to epithelium in the group filled with the nCSi@3G3Si hierarchical architectures confirmed that the 3G3Si hydrogel unit can prevent the infiltration of epithelial cells. Further evidence was also observed when longer PDL was formed in the biphasic architecture group. In the nCSi group, the bioceramic scaffold resisted the migration of the oral epithelium to root surface at 4 weeks, whereas the epithelium still approached the base of the defects after 8 weeks. These results verify the potent potential of 3G3Si hydrogel to protect periodontal defects from epithelium tissue invasion. In this regard, the barrier efficacy of Si-HPMC component has been confirmed in previous studies [52, 61]. That is, the penetration of epithelium cells could be strongly inhibited by the Si-HPMC hydrogel.

As for the potential clinical manageability, the 3G3Si hydrogel membrane was crosslinked on the top side wall of customized nCSi scaffolds for fabricating the modularized biphasic architectures with personalized outward morphology (Fig. 6). It is noted that when the gelation is finished in the interconnected pores of the bioceramic scaffolds, the migration and colonization of the host cells for periodontal regeneration may be compromised. Based on the rheological parameters and mechanical properties, the 3G3Si hydrogel indicated a suitable physiochemical properties and favorable biological activity. Thus, the nCSi@3G3Si biphasic architecture was fabricated and used in a single-step GTR procedure, whereby the hierarchical architecture can act as both a barrier and a bone graft. Evidently, the stiffness of the nCSi scaffold may prevent the collapse of hydrogel membrane, providing a stable environment for host cells colonization. In fact, the periodontal multi-tissue engineering is challenging due to the complex interaction of multiple soft and hard tissues [62]. So, it is ideally suitable to applied a biphasic scaffold approach in periodontal tissue engineering [63, 64]. In 2014, Requicha et al. developed a double-layer scaffolds based on a combination of a 3D fiber mesh functionalized with silanol groups aiming at promoting bone formation and a barrier membrane, both made of starch and poly-e-(caprolactone) (SPCL) [63]. Although the proposed double-layer scaffold can support the proliferation and selectively promotes the osteogenic differentiation of canine adipose stem cells (cASCs) seeded on the functionalized mesh, no in vivo studies were performed. More recently, a two-layered bioactive membrane were fabricated whereas one layer was selected for bone regeneration and produced using poly glycerol sebacate (PGS)/PCL and β-TCP, and another layer containing PCL/PGS and chitosan acted as barrier membrane [65]. The appropriate osteoconductivity, flexibility along with barrier properties of this biphasic dental membrane were proved in vitro. However, such previous scaffold architectures did not replicate the anatomy of the defects or perform in vivo studies in periodontal defect models. In our study, the personalized architectures can be implanted in the defects directly, without bone grafts shaping, barrier membrane tailoring or fixation (Fig. 7A). It is evident that the technical sensitivity of the GTR surgery is reduced and the time of operation can be saved.

Finally, the periodontal regeneration potential of the biphasic architecture was evaluated by a dog periodontal defect model. μCT reconstruction (Fig. 7B) proved that the hydrogel membrane can create a protective environment for periodontal bone tissue regeneration and induce alveolar bone forming. Histological analysis (Figs. 8 and 9) further confirm that the personalized biphasic hierarchical architectures displayed an expected periodontal reconstruction predictability in the challenging one-wall intrabony defects. On the one hand, hydrogel membrane can isolate the defects from epithelial and connective tissue for re-establishing functional periodontal tissue. Though the hydrogel membrane was not found in histological staining, the barrier effect was confirmed by MacNeal staining and quantitative analysis (Fig. 9). The hydrogel biodegradation may be ascribed to the long-time stage in vivo, and the membrane has been resorbed almost completely. It is acknowledged that the structure of barrier membrane must be maintained in vivo for more than two weeks, the length of time critical for soft tissue migration [26]. On the other hand, the nCSi scaffolds are served as the microporous constructs for conducting and stimulating alveolar bone tissue regeneration. It is reported that calcium, magnesium and silicon ions released from the nCSi scaffolds have significant osteogenic activity and magnesium ions can also promote the proliferation and differentiation of pre-osteoblasts [66, 67]. Meanwhile, the osteogenesis of the porous architecture can be enhanced by hydrogel membrane according to the studies in vitro. So, the process of regeneration in intrabony defects may be proposed as follows. First, the epithelial and gingival connective tissue are blocked by hydrogel membrane from root surface. Then, progenitor cells and/or stem cells from PDL recolonize the wound supported by the space-providing porous nCSi scaffold. Finally, progenitor cells differentiate into new fibroblasts, cementoblasts and osteoblasts that responsible for periodontal regeneration. In the meantime, the regeneration of alveolar bone can be enhanced by nCSi scaffold and hydrogel membrane though promoting the migration and differentiation of osteoblasts. However, this one-step GTR approach solely relied on the regenerative performance of the host cells residing nearby or migrating into the damaged area. Because the GelMA and Si-HPMC hydrogels have unique advantages for controlled release of bioactive substance, cell seeding and encapsulation [39, 55, 68–70], the in vivo outcomes of this biphasic hierarchical architecture may be improved by combining with a cell-based or drug-delivery approach in the future.

## Conclusions

In summary, we have developed a functional biphasic hierarchical architecture comprising of nCSi porous scaffolds and GelMA/Si-HPMC membrane with finely tuned chemical compositions, complex shape and inner architectures based on alveolar bone anatomy for periodontium reconstruction. The nCSi scaffolds was fabricated to match the alveolar bone defects, and the hydrogel membrane was formed in the personalized mold to match the outward structure of bioceramic scaffold and periodontal defects. The hydrogel membranes could tightly integrate with the porous scaffolds with a controlled biodegradation and exhibited favorable biocompatibility, osteogenesis and barrier effect in vitro. Meanwhile, the porous hierarchical architectures exhibited a strong predictability of periodontal regeneration in the periodontal intrabony defect models, which may be favorable for significantly reducing the technical sensitivity of the operation. These findings suggested that such pro-designed hierarchical architectures have excellent potential for future periodontal regeneration in clinic.

## Data Availability

Not applicable.
